# Enhancing Stability and Bioavailability of Peptidylglycine Alpha-Amidating Monooxygenase in Circulation for Clinical Use

**DOI:** 10.3390/biom15020224

**Published:** 2025-02-04

**Authors:** Yulia Ilina, Paul Kaufmann, Michaela Press, Theo Ikenna Uba, Andreas Bergmann

**Affiliations:** 1 PAM Theragnostics GmbH, 16761 Hennigsdorf, Germany; 2 4TEEN4 Pharmaceuticals GmbH, 16761 Hennigsdorf, Germany

**Keywords:** peptidylglycine alpha-amidating monooxygenase, C-terminal amidation, peptide hormones, pharmacokinetics, half-life extension

## Abstract

Peptidylglycine alpha-amidating monooxygenase (PAM) is the only enzyme known to catalyze C-terminal amidation, a final post-translational modification step essential for the biological activity of over 70 bioactive peptides, including adrenomedullin (ADM), calcitonin gene-related peptide (CGRP), amylin, neuropeptide Y (NPY), and others. Bioactive (amidated) peptide hormones play crucial roles in various physiological processes and have been extensively explored as therapeutic compounds in clinical and preclinical research. However, their therapeutic viability is limited due to their short half-life and, in most cases, the need for prolonged infusion to maintain effective concentrations. PAM itself has also been considered as a therapeutic compound aiming to increase the level of amidated peptide hormones; however, similarly to peptide hormones, PAM’s rapid degradation limits its utility. Here, we present a strategy to enhance PAM stability and bioavailability through PEGylation, significantly extending the enzyme’s half-life in circulation assessed in healthy rats. Furthermore, single subcutaneous (s.c.), intramuscular (i.m.), or intraperitoneal (i.p.) administration of PEGylated PAM resulted in a sustained increase in circulating amidating activity, with peak activity observed at 12–24 h post-bolus administration. Notably, amidating activity remained significantly elevated above baseline levels for up to seven days post-administration, with no observable adverse effects. These findings highlight PEGylated PAM’s potential as a viable therapeutic compound.

## 1. Introduction

Peptidylglycine α-amidating monooxygenase (PAM) is a bifunctional enzyme crucial for the C-terminal amidation of peptide hormones—a post-translational modification essential for the biological activity of nearly half of all known peptides. This modification, uniquely catalyzed by PAM, enhances peptide receptor affinity, stabilizes peptides against proteolytic degradation, and is indispensable for their full biological functionality [1,2]. Among the many peptides requiring amidation are adrenomedullin (ADM), calcitonin gene-related peptide (CGRP), amylin, neuropeptide Y (NPY), substance P, pituitary adenylate cyclase-activating polypeptide (PACAP), vasoactive intestinal peptide (VIP), and many others [2,3,4] (Figure S1).

The amidation process requires the presence of a C-terminal glycine on the peptide substrate. PAM carries out this reaction in two sequential steps mediated by its distinct functional domains. First, the peptidylglycine α-hydroxylating monooxygenase (PHM) domain hydroxylates the C-terminal glycine. Next, the peptidyl-α-hydroxyglycine α-amidating lyase (PAL) domain removes glyoxylate, producing a C-terminal amide [1].

Dysregulation of amidated peptide hormones has been associated with multiple pathologies, including neurodegenerative diseases, cardiovascular conditions, metabolic disorders, and many others [5,6,7,8,9,10]. The idea of using such peptide hormones as therapeutic compounds is not new, and several attempts have been made in this respect within preclinical and clinical studies. ADM is a multifunctional peptide hormone with potent vasodilatory, anti-inflammatory, and angiogenic properties, playing a critical role in cardiovascular homeostasis and immune regulation. ADM has demonstrated therapeutic potential in inflammatory bowel disease (e.g., refractory ulcerative colitis), cardiovascular diseases (heart failure, myocardial infarction, pulmonary hypertension), vascular cognitive impairment, ischemic stroke, and sepsis [11,12,13,14,15]. VIP displays neuroprotective, anti-inflammatory, and immunomodulatory effects, making it promising for conditions such as Parkinson’s, Alzheimer’s disease (AD), and autism spectrum disorders, as well as brain injuries [5,6,16]. VIP also showed relevance for treating pulmonary disorders (asthma and COPD) [17] and autoimmune diseases (rheumatoid arthritis, multiple sclerosis) [18]. PA-CAP offers neuroprotection and potentiates as a biomarker for neurological diseases such as Alzheimer’s, Parkinson’s, and multiple sclerosis [19,20,21,22]. PACAP also shows promise in treating retinal degenerations of metabolic origin and rescuing synaptic plasticity in fragile X syndrome [23,24]. In Alzheimer’s disease preclinical models, PACAP administration slows down pathology progression, improves cognitive function, and protects against beta-amyloid toxicity [25,26]. Amylin, a pancreatic hormone, has emerged as a potential therapeutic agent for AD and type 2 diabetes (T2D) due to its role in glucose homeostasis and neuroprotection [27]. Studies have shown that amylin and its analogs, such as pramlintide, can reduce AD pathology by decreasing amyloid-β, phospho-tau, and inflammation in animal models [28]. Amylin treatment has also been found to improve cognitive function in AD mouse models [28].

As summarized above, the direct administration of peptide hormones has been explored as a strategy to mitigate disease progression, but its therapeutic applications re-main limited. Subcutaneous administration and inhalation have been explored for peptide hormone delivery, but their success is limited to a few hormones [29,30]. Prolonged intra-venous infusion remains the most common method to elevate peptide levels due to their rapid degradation and short half-life [12]. Additionally, gradual adjustment of peptide concentration achieved via infusion is required to prevent adverse effects, such as systemic vasodilation, hypotension, aggregation, etc., which could result from a rapid rise in peptide levels if bolus administration is used [12,31].

Along with dysregulation of peptide hormones, changes in PAM levels have been associated with the emergence or onset of diverse pathologies [32]. Patients suffering from multiple endocrine neoplasia type 1 and pernicious anemia showed a decreased plasma PAM activity in comparison to healthy control subjects [33]. The LoF mutations of PAM have been associated with an increased risk of type 2 diabetes and sarcopenic diabetes, potentially through impacts on insulin granule packaging and secretion from β-cells [34,35,36]. Notably, reduced PAM activity, when measured in the cerebrospinal fluid of Alzheimer’s patients, was significantly lowered compared to that in healthy individuals [37]. Beyond its primary role, Bäck et al. have shown PAM’s necessity in the formation of atrial secretory granules [38]. Together, these findings underscore PAM’s pivotal role in regulating various physiological and pathophysiological processes, either by modulating peptide hormone levels or serving as a therapeutic compound itself.

Building on this premise, we explored the strategy of enhancing circulating PAM levels to indirectly increase the concentration of amidated peptide hormones by supporting endogenous hormone maturation. Previous work by Kaufmann et al. in 2021 demonstrated that the intravenous bolus administration of unmodified PAM temporarily elevated amidating activity and the circulating levels of amidated adrenomedullin in rats, but its therapeutic potential was constrained by a plasma half-life of only 47 min [39]. To address these limitations, we PEGylated PAM, significantly improving its stability and extending its circulatory half-life to approximately 218 min in rats. PEGylated PAM also showed elevated amidating activity across subcutaneous, intramuscular, and intraperitoneal routes, with peak activity increasing 900–1800% within 12–24 h of a single bolus (225 µg/kg animal weight) in circulation and sustained elevation lasting up to seven days. Safety assessments indicated that PEGylated PAM was well tolerated, with no adverse effects on behavior, weight, or physiological patterns observed. Furthermore, its pharmacokinetic profile, with a lower Cmax and higher AUC than unmodified PAM, may reduce the risk of adverse effects associated with rapid hormone spikes. Finally, this approach provides a stable, clinically viable method to enhance endogenous hormone maturation, offering potential therapeutic benefits for conditions associated with dysregulated peptide hormone levels and/or impaired PAM function.

## 2. Materials and Methods

### 2.1. PAM Constructs and PEGylation

For all animals, experiment recombinant human PAM enzyme expressed in HEK293 cells, covering the full-length sequence from Met1 to Ser866 and purchased from SinoBiological (catalog number: 13624-H08H; NP_620176), was used. The resulting constructs are both soluble variants homologous to isoform 3 but exclude the protease-sensitive linker region encompassing amino acids 388 to 494. PAM enzyme was pegylated according to the following PEGylation protocol. Briefly, 10 mg lyophilized PAM was dissolved in 5 mL phosphate-buffered saline (PBS, pH 10). A 20 mM solution of PEG-5000 (MeO-PEG-NHS, Iris Biotech, Marktredwitz, Germany) was prepared by dissolving in 20% DMSO and added to the PAM solution at a 90–120-fold molar excess, equating to a 2.2-fold excess relative to lysine residues within PAM. The mixture was incubated for 180 min at 4 °C with agitation at 100 rpm and quenched subsequently with 500 µL of 1 M unbuffered Tris solution. Separation of PEGylated PAM (PEG-PAM) from free PEG was achieved through size-exclusion chromatography (Superdex 200 16/600-pg, Cytiva Europe GmbH, Freiburg, Germany) with PBS as eluent. Fractions indicative of molecular weights between 160 and 90 kDa were pooled, measured for the amidating activity using the methods described elsewhere [39], sterile-filtered through a 0.2 µm filter, aliquoted, and stored at −80 °C for in vivo application.

### 2.2. PAM In Vivo Pharmacokinetics

A single intravenous (i.v.) dose of PEG-PAM or unmodified PAM, adjusted to a dose of 72 µg/kg of body weight (approximately 28.8 µg per rat and 2 Units per rat) at 10 mL/kg, was administered for pharmacokinetic assessment. Six male Wistar rats (2.5–3 months old, ≥350 g) were divided into two groups with three animals per group (group A and group B). For group A, blood samples (300 µL whole blood) were collected into Li-heparinized tubes from lateral tail vein under 5 vol.% isofluran at predetermined intervals: pre-administration and at 20, 60, 120, 240, and 300 min post-injection. For group B, blood samples were collected in the same manner as for group A at predetermined intervals: pre-administration and at 40, 80, 180, 240, and 300 min post-injection. Animals were awake between the different blood sampling time points. The samples were processed within 5 min after the blood withdrawal and stored as Li-heparin plasma at −80 °C until further analysis. This animal study was conducted by preclinics Gesellschaft für Präklinische Forschung GmbH (Potsdam, Germany) and the study protocol was approved by the State Office for Occupational Safety, Consumer Protection, and Health of the State of Brandenburg (protocol number 2347-48-2017, sub-experiment 14/21, date of approval 20 March 2018). The animals used in this study were obtained from Charles River Laboratories, Sulzfeld, Germany.

A single subcutaneous (s.c.), intramuscular (i.m.), or intraperitoneal (i.p.) dose of PEG-PAM adjusted to 225 µg/kg body weight (approximately 90 µg per rat and 6 Units per rat) was administered to Sprague Dawley rats with six animals per group. For s.c. and i.p. administrations, the volume applied was 10 mL/kg, while for i.m. administration the volume was fixed at 200 µL per animal. Blood samples (300 µL whole blood) were collected in Li-heparinized tubes under isoflurane anesthesia. Sampling was performed retrobulbarly from the V. saphena and from the V. jugularis at predetermined intervals: pre-administration and at 15 min, 30 min, 60 min, 2 h, 4 h, 8 h, 12 h, 24 h, 3 days, and 7 days post-administration. The sampling was alternated between left and right sides. Samples were processed within 20 min of collection and stored as Li-heparin plasma at −80 °C until further analysis. Rats were anesthetized prior to substance administration and remained anesthetized throughout the experiments. This animal study was conducted in accordance with the German Animal Welfare Act and European Council Directive 86/609/EEC by Bioassay Labor für biologische Analytik GmbH (Heidelberg, Germany) and the study protocol was approved by the Regional Council of Karlsruhe (protocol number 35-9185.81/G192/17, date of approval 26 October 2017). The animals used in this study were obtained from Charles River Wiga GmbH, Sulzfeld, Germany.

**Figure 1:**
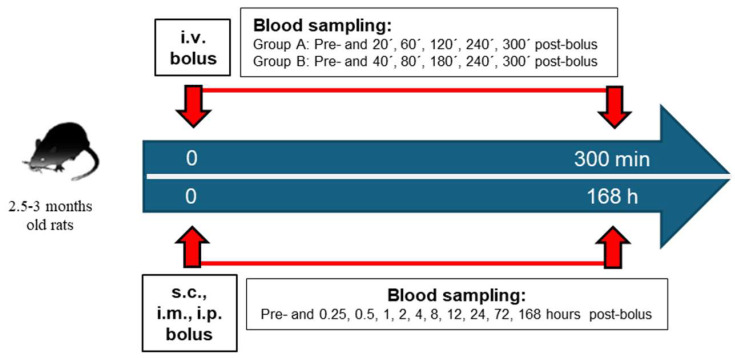
Schematic representation of the experimental setup for assessing the pharmacokinetics and the enrichment of amidating activity of PAM enzyme in circulation after intravenous (i.v.), subcutaneous (s.c.), intramuscular (i.m.), and intraperitoneal (i.p.) administration. The details of this study are described in the Section 2.

The amidating activity was measured as previously described by Kaufmann et al. (2021) [39]. Specific PAM activity was calculated by dividing the measured PAM activity by the PAM quantity, determined using the bicinchoninic acid (BCA) protein assay according to the manufacturer’s protocol. Non-compartmental analysis was performed using Phoenix WinNonlin version 8.4 (Certara USA, Inc., Princeton, NJ), calculating the Tmax, Cmax, and AUC. Half-life (t1/2) was determined independently in both groups and the average t1/2 was reported. The activity at each time point was derived by subtracting the mean baseline (t = 0) from the mean measurement at each timepoint. The schematic representation of the study design for both in vivo experiments is shown in Figure 1.

## 3. Results

To enhance PAM stability, we performed PEGylation. Figure S2A shows that the activity of PEGylated PAM was minimally reduced compared to that of non-modified PAM, with a reduction in activity of 4.5% (coefficient of variation (CV)). The PEGylation resulted in a molecular weight shift from approximately 90 kDa to approximately 130 kDa (Figure S2B), indicating successful PEGylation, as well as covalent attachment of PEG to PAM.

**Figure 2:**
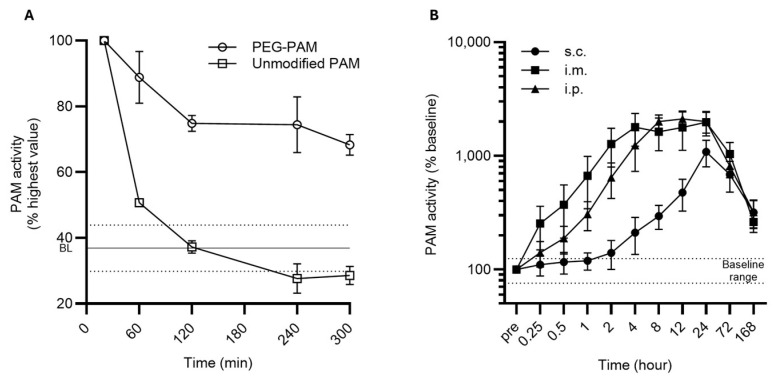
(**A**) Time-resolved decay of relative amidating activity between PEG-PAM and unmodified PAM following intravenous (i.v.) bolus administration (group A). The amidating activity at the 20 min post-bolus time point was set as 100%, with all other values expressed relative to this peak activity. The CV was calculated for the relative baseline values and the CV value was then used to define the upper and lower bounds of the baseline range (dashed lines). BL (solid line)—mean relative baseline value; (**B**) circulating relative amidating activity of PEG-PAM following subcutaneous (s.c.), intramuscular (i.m.), and intraperitoneal (i.p.) administration. The pre-bolus activity values were set to 100%. The CV value was then used to establish the upper and lower bounds of the baseline range (dashed line).

The basic pharmacokinetic parameters are summarized in Table 1 and Table S1. The half-life time for PEG-PAM and unmodified PAM was calculated by measuring the half-life in circulation after i.v. bolus administration. To limit blood withdrawal frequency, animals were subdivided into two groups (group A and B) with time-shifted blood withdrawal frequencies as described in Section 2. The baseline amidating activity averaged across all groups was 12.7 × 10^3^ ± 2.5 × 10^3^ Units (mean ± standard deviation (SD)). The PEGylation extended the in vivo half-life of PEG-PAM to 223.8 min for group A and to 212.7 min for group B leading to an average 218.2 min, while the mean half-life of unmodified PAM was only 42.3 min (Figure 2A; Table 1 and Table S1), consistent with previous reports [39], where the half-life of unmodified PAM in circulation was 47 min. In the case of unmodified PAM, the amidating activity dropped within the baseline range after 100 min after reaching the peak activity (Figure 2A and Figure S3). In contrast, PEG-PAM maintained elevated activity for ca. 6 h post-administration, retaining over 65% of the peak activity in groups A and B (Figure 2A and Figure S3). Additionally, the PEGylation of PAM resulted in a higher AUC (80.9 × 10^3^ h*Units for PEG-PAM compared to 38.3 × 10^3^ h*Units for unmodified PAM (Table 1)) and lower Cmax (18.3 × 10^3^ Units for PEG-PAM vs. 27.9 × 10^3^ Units for unmodified PAM).

Figure 2B illustrates that PEG-PAM administered via s.c., i.p., and i.m. routes led to prolonged and elevated amidating activity in circulation, surpassing levels observed with i.v. bolus administration. The baseline amidating activity averaged across all groups was 6.1 × 10^3^ ± 1.5 × 10^3^ Units (mean ± standard deviation (SD)). The individual baseline levels of amidating activity and further pharmacokinetic parameters per application route are reported in Table 1. Following s.c. administration, PAM activity reached a maximum of 50.2 × 10^3^ Units at 24 h post-bolus, representing a 835% increase from the baseline (5.4 × 10^3^ ± 1.2 × 10^3^ Units); this activity remained elevated at 204% above the baseline at 168 h post-administration. For i.m. administration, PAM activity peaked at 93.9 × 10^3^ Units (an approximate 1200% increase) after 24 h, with sustained activity at 90% above the baseline 168 h post-PEG-PAM administration. The i.p. route produced the highest peak activity of 104.6 × 10^3^ Units (ca. 1730% increase) at 12 h, with levels still 180% above the baseline at 168 h post-PEG-PAM administration. In terms of the AUC, i.p. administration yielded the highest exposure (7.0 × 10^6^ h*Units), followed by i.m. (6.5 × 10^6^ h*Units), with s.c. showing the lowest total exposure (4.2 × 10^6^ h*Units).

No adverse effects were observed in all PAM-treated animals. All animals maintained good clinical condition, displaying normal social interactions with peers, appropriate grooming behavior, regular drinking and eating habits, as well as normal weight development. Sleep patterns also remained unaffected.

## 4. Discussion

This study presents a comprehensive approach to enhancing circulating amidating activity and substantially increasing the bioavailability of PAM. PEGylation significantly improved the stability and circulatory half-life of PAM, as evidenced by prolonged amidating activity across all tested administration routes (i.v., s.c., i.p., and i.m.).

The pharmacokinetic analysis revealed that PEG-PAM had a markedly extended half-life of approximately 218 min following intravenous administration, compared to 42 min for unmodified PAM. However, this estimate may be conservative, as amidating activity was still elevated above the baseline during the observation period of 6 h post-bolus, suggesting that the actual half-life may be longer. The lower Cmax and higher AUC observed with PEG-PAM indicate a slower systemic release, potentially mitigating the risks associated with abrupt hormone spikes, such as systemic vasodilation from sudden increases in amidated peptide hormones like adrenomedullin. The comparison of pharmacokinetic parameters (Cmax, Tmax, and AUC) across non-intravenous routes demonstrated that all the tested routes of administration effectively increased endogenous amidating activity and systemic exposure. Subcutaneous administration, in particular, offers a promising route for therapeutic applications due to its practicality for self-administration.

An important question to address is whether PEGylated PAM retains its enzymatic activity in vivo, given that ex vivo measurements of amidating activity using the PAM-AMA assay are conducted under optimal conditions, including an optimal pH range and optimal cofactor concentrations (5 µM copper and 2 mM ascorbate). These conditions differ from the physiological environment in circulation. The optimal pH for PAM activity aligns with the acidic environment of secretory granules (pH 5.0–5.5), which is different from the more basic pH of blood (~7.4). Nonetheless, in the amidating assay previously reported by Kaufmann et al., 2021 [39], conducted at pH 7.5 to approximate physiological conditions, we observed the conversion of glycine-extended adrenomedullin (ADM-Gly; a PAM substrate) into bioactive adrenomedullin (bio-ADM; product of amidation by PAM). This indicates that PAM retains significant enzymatic activity even under non-optimal pH conditions. Furthermore, the retention of the enzymatic activity following exogeneous PAM administration can be inferred from changes in the levels of amidated peptides, such as, e.g., adrenomedullin. As previously demonstrated for unmodified PAM [39], increases in circulating PAM activity correlate well with changes in relative bio-ADM concentrations, even in the absence of exogenous ascorbate. Similarly, after PEG-PAM administration, we observed a consistent trend: elevated PAM activity in circulation was associated with a corresponding increase in bio-ADM levels (Figure S4). However, due to considerable variability among individual rats and resulting large standard deviations, statistical significance was not reached at most time points. Despite this limitation, the observed trend strongly suggests that PEGylated PAM retains enzymatic activity in vivo in circulation after delivery, pointing to the value of further investigation with larger group sizes.

The literature indicates that amidation primarily occurs intracellularly within secretory granules, with mature peptides being released into circulation. However, this reaction does not achieve 100% efficiency. For example, studies have shown that adrenomedullin exists predominantly in its inactive glycine-extended form in circulation, ranging between the 5.6:1 and 2:1 ratios of glycine-extended to bioactive forms in healthy individuals [40,41]. This implies that inactive substrates for PAM are already present in circulation, allowing PEG-PAM to function effectively without the risk of overdosing or excessive hormone activation, enhancing its safety profile. Concerns that PAM activity might be limited to intracellular environments were previously addressed by Kaufmann et al. in 2021 [39], who demonstrated that amidation can also occur in circulation. This finding supports the feasibility of enhancing systemic PAM activity to modulate peptide hormone levels therapeutically.

We observed the differences in baseline amidating activity between experiments involving different administration routes. Animals receiving PEG-PAM intravenously exhibited significantly higher baseline activity compared to those receiving subcutaneous, intramuscular, or intraperitoneal injections. This discrepancy may stem from variations in blood sampling methods; for intravenous administration, blood was collected from the lateral tail vein, whereas for other routes retrobulbar sampling and sampling from V. saphena and V. jugularis were used. Such variations, coupled with the unknown primary source of circulating PAM, could explain the observed differences. Additionally, the use of different rat strains across experiments may have contributed to baseline variability.

Importantly, no adverse effects were detected throughout this study. Animals exhibited normal physiological behaviors, including social interactions, grooming, weight maintenance, and sleep patterns. This favorable safety profile, combined with extended systemic exposure and sustained activity, highlights PEG-PAM’s potential as a therapeutic agent for conditions requiring long-term modulation of amidated peptide levels. Nonetheless, comprehensive toxicity and safety assessments remain necessary for further development.

One concern in this study is the use of a His-tagged recombinant protein, which could increase immunogenicity, particularly in the context of long-term PAM applications. Nonetheless, this study establishes a proof of principle, demonstrating for the first time the therapeutic potential of PAM to enhance circulating amidating activity and increase levels of amidated peptide hormones with clinical relevance. For future clinical use, particularly in chronic conditions, the development of an untagged construct will be critical to minimize such immunogenicity concerns, as well as to avoid potential interference of the His-tag with amidating activity, as the His-tag has the capacity to bind copper, an essential cofactor for PAM’s enzymatic function. Another significant concern is the broad substrate specificity of PAM, which can activate a wide range of peptide hormones with diverse effects. While an elevation in certain peptide hormones has been shown to have beneficial effects, an increase in others can lead to deleterious outcomes. An increase in bio-ADM supports vascular health by promoting endothelial repair and maintaining vascular homeostasis [42], while elevated GLP-1 enhances insulin secretion, improves glucose regulation, and aids in weight management [43]. Similarly, higher PACAP levels provide neuroprotection by reducing neuronal cell death and offering therapeutic potential in neurological disorders [44]. Likewise, increased amylin contributes to blood glucose control by inhibiting glucagon [45]; cholecystokinin enhances digestion, supporting long-term metabolic balance [46]; and vasopressin helps regulate blood pressure and renal function [47]. Conversely, elevated substance P and CGRP are associated with migraines and pain sensitization [48], while high NPY levels may contribute to obesity and metabolic syndrome [49]. Excessive vasopressin can lead to water retention disorders [50], and chronically elevated gastrin has been linked to hyperplasia and an increased cancer risk [51]. Therefore, before progressing to human applications, the utilization of the drug candidate in regulatory tox and safety studies is mandatory to establish a detailed safety profile. Furthermore, it will be critical to stratify eligible patients by assessing levels of active and inactive peptide hormones (where possible), as well as PAM levels in various pathological conditions, to create a risk–benefit assessment for the target pathology of the treatment. Currently, to our knowledge, adrenomedullin is the only peptide hormone for which both active (bio-ADM) and inactive precursor (ADM-Gly) forms can be directly measured using immunoassays, which are validated and applicable for large-scale human sample processing [40,52]. Developing similar immunoassays for other peptide hormones with various therapeutical properties, e.g., VIP and NPY, would greatly enhance patient classification and help mitigate potential risks associated with PAM therapy.

In summary, PEG-PAM administered via non-intravenous routes achieves prolonged systemic effects without adverse outcomes. These findings lay the groundwork for further studies to explore its long-term pharmacodynamic profile, therapeutic efficacy, and potential applications across diverse disease models.

## 5. Conclusions

Taken together, our data provide a robust strategy to significantly enhance the stability and bioavailability of PAM, a critical enzyme for the C-terminal amidation of peptide hormones—a reaction essential for their full biological activity. By extending the circulatory half-life of PAM through PEGylation, we demonstrate a novel approach to achieving prolonged systemic amidating activity via subcutaneous, intramuscular, and intraperitoneal administration routes. Remarkably, a single bolus application sustained amidating activity above baseline levels for over a week, with no observed adverse effects. This method minimizes the risks associated with overdosing amidated peptide hormones by utilizing the naturally inactive glycine-extended peptide precursors already present in circulation as substrates. These findings suggest that PEGylated PAM offers a stable, clinically viable solution to enhance endogenous hormone maturation, providing potential therapeutic benefits for conditions associated with dysregulated peptide hormone levels and impaired PAM function. Further studies are needed to explore the long-term pharmacodynamic profile and therapeutic efficacy of PEG-PAM across diverse disease contexts.

## 6. Patents

This work has resulted in a patent application under international publication number WO 2021/170816 A1 (international application number PCT/EP2021/054869).

## Figures and Tables

**Table 1:** Pharmacokinetic parameters of PEG-PAM and unmodified PAM following intravenous (i.v.), intramuscular (i.m.), intraperitoneal (i.p.), or subcutaneous (s.c.) bolus administration in rats. Key metrics include t1/2 (half-life), Cmax (maximum concentration in plasma), area under the curve (AUC), baseline (BL) and peak activity, as well as the amidating activity 7 days post-bolus PAM injection; * describes the mean value between groups A and B.

Route	Parameter		PEG-PAM	Unmodified PAM
	Variable	Units		
i.v.	Animals	n/group	6	6
	T1/2 *	min	218.2	42.3
	Cmax *	Units	18.3 × 10^3^	27.9 × 10^3^
	AUC *	h*Units	80.9 × 10^3^	38.3 × 10^3^
	BL activity	Units (mean + SD)	12.7 × 10^3^ ± 2.8 × 10^3^	12.7 × 10^3^ ± 2.3 × 10^3^
i.m.	Animals	n/group	6	
	Cmax	Units	93.9 × 10^3^	
	Tmax	h	24	
	AUC	h*Units	6.5 × 10^6^	
	BL activity	Units (mean ± SD)	7.2 × 10^3^ ± 1.8 × 10^3^	
	7d post-bolus activity		13,615 ± 1370	
i.p.	Animals	n/group	6	
	Cmax	Units	104.6 × 10^3^	
	Tmax	h	12	
	AUC	h*Units	7.0 × 10^6^	
	BL activity	Units (mean ± SD)	5.7 × 10^3^ ± 0.8 × 10^3^	
	7d post-bolus activity		16.2 × 10^3^ ± 2.0 × 10^3^	
s.c.	Animals	n/group	6	
	Cmax	Units	50.2 × 10^3^	
	Tmax	h	24	
	AUC	h*Units	4.2 × 10^6^	
	BL activity	Units (mean ± SD)	5.4 × 10^3^ ± 1.2 × 10^3^	
	7d post-bolus activity		16.3 × 10^3^ ± 2.1 × 10^3^	

## Data Availability

The original contributions presented in this study are included in the article/Supplementary Materials. Further inquiries can be directed to the corresponding author.

## Supplementary Materials

The following supporting information can be downloaded at https://www.mdpi.com/article/10.3390/biom15020224/s1, Figure S1: Maturation pathway of peptide hormones; Figure S2: PEGylation of PAM enzyme, Figure S3: Time-resolved decay of relative amidating activity between PEG-PAM and unmodified PAM following intravenous (i.v.) bolus administration (group B); Figure S4: Circulating relative amidating activity of PEG-PAM following intraperitoneal administration and the relative change in bio-ADM concentration; Table S1: Pharmacokinetic parameters of PEG-PAM and unmodified PAM following intravenous bolus administration in rats in group A and group B; Supplementary File S1: Original images for Figure S2B.

## Abbreviations

The following abbreviations are used in this manuscript:

PAM	peptidylglycine alpha-amidating monooxygenase
ADM	adrenomedullin
CGRP	calcitonin gene-related peptide
NPY	neuropeptide Y
VIP	vasoactive intestinal peptide
PACAP	pituitary adenylate cyclase-activating polypeptide
T2D	type 2 diabetes
COPD	chronic obstructive pulmonary disease
CV	coefficient of variation
AUC	area under the curve
Cmax	maximum analyte concentration in plasma
Tmax	time to reach maximum analyte concentration
BL	baseline
i.v.	intravenous
i.m.	intramuscular
i.p.	intraperitoneal
