# Supplementary material for: Enhancing Stability and Bioavailability of Peptidylglycine Alpha-Amidating Monooxygenase in Circulation for Clinical Use
Source: Biomolecules. 2025 Feb 4;15(2):224. doi: 10.3390/biom15020224 (PMC11853079; doi:10.3390/biom15020224)
Supplement: Supplementary file 1 [file biomolecules-15-00224-s001.zip › biomolecules-3405411-supplementary/biomolecules-3405411-supplementary.pdf]

Supplementary

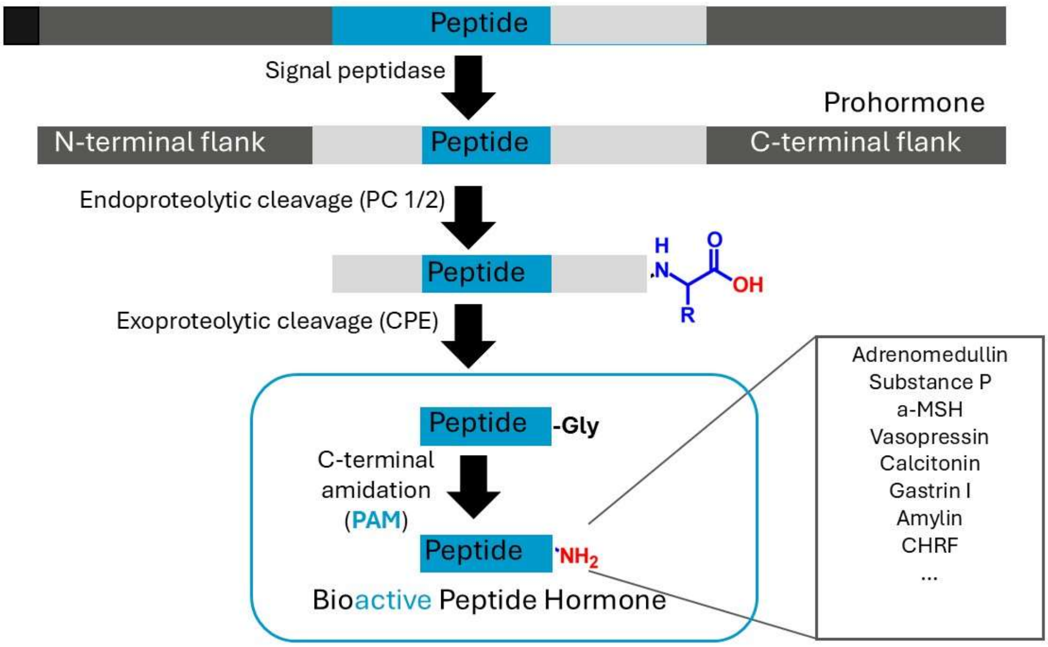

Figure S1: Maturation pathway of peptide hormones.

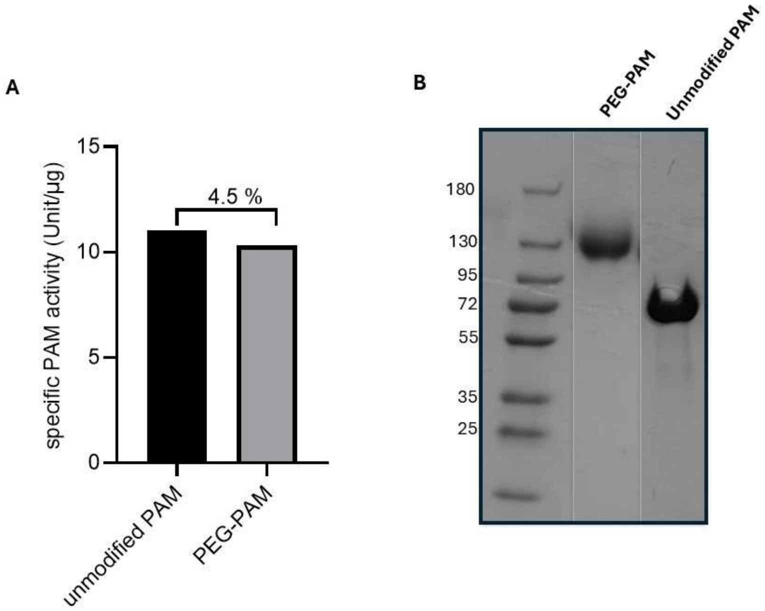

Figure S2: PEGylation of PAM enzyme. (A) Comparison of specific enzymatic activity between PEGylated and unmodified PAM; 4.5% (CV) reduction in activity following PEGylation. (B) SDS-PAGE showing a molecular weight increase from ~90 kDa to ~130 kDa, confirming successful PEGylation. The original image can be found in Supplementary File 1.

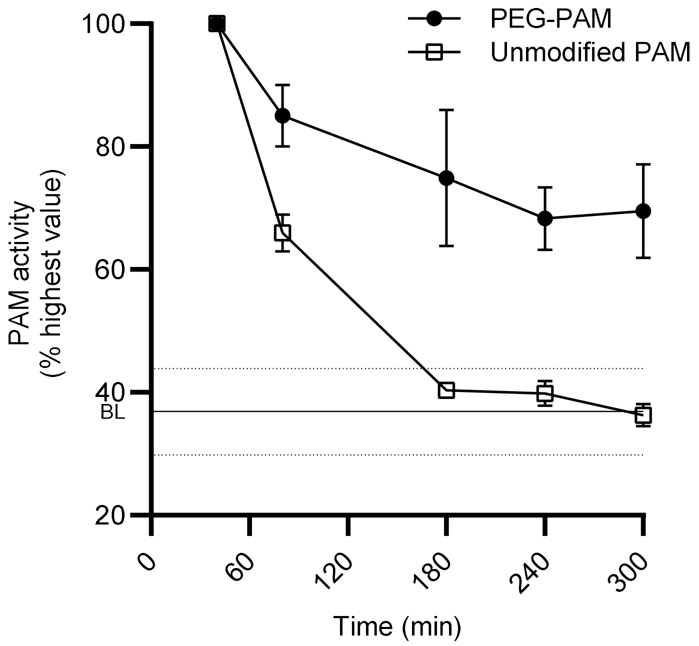

Figure S3: Time-resolved decay of relative amidating activity between PEG-PAM and unmodified PAM following intravenous (i.v.) bolus administration (group B). The amidating activity at the 40-minute post-bolus time point was set as 100%. The CV was calculated for the relative baseline values and the CV value was then used to define the upper and lower bounds of the baseline range (dashed lines). BL – mean relative baseline value.

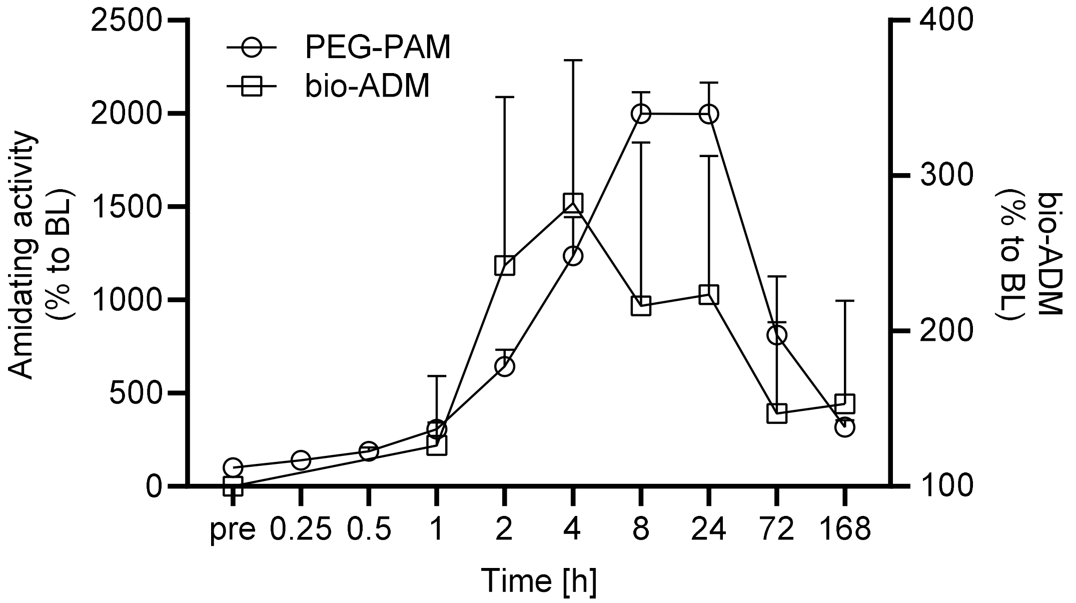

Figure S4. Circulating relative amidating activity of PEG-PAM following intraperitoneal administration and the relative change in bio-ADM concentration. Pre-bolus amidating activity and bio-ADM concentration values were normalized to 100%. Bio-ADM concentrations were measured using a commercially available assay as described by Weber et al., 2017 (n = 4) [52]. Two rats were excluded from the analysis due to bio-ADM concentrations being below the limit of detection in most samples. Error bars represent standard deviation.

Table S1: Pharmacokinetic parameters of PEG-PAM and unmodified PAM following intravenous bolus administration in rats in group A and group B. Key metrics include t1/2 (half-life time), Cmax (maximum concentration in plasma), Tmax (time to reach maximum concentration in plasma), area under the curve (AUC), baseline and peak amidating activity.

Route	Parameter		PEG-PAM		unmodified PAM	
	Variable	Units	Group A	Group B	Group A	Group B
i.v.	Animals	n/group	3	3	3	3
	T1/2	min	223.8	212.7	53.8	30.8
	Cmax	Units	17.0×10^3	19.7×10^3	38.1×10^3	17.7×10^3
	Tmax	min	20	40	20	40
	AUC	h*Units	75.8×10^3	86.0×10^3	45.7×10^3	30.9×10^3
	Baseline activity	Units (mean + SD)	$10.9 \times 10^3 \pm 1.7 \times 10^3$	$14.5 \times 10^3 \pm 2.7 \times 10^3$	$14.2 \times 10^3 \pm 1.8 \times 10^3$	$11.2 \times 10^3 \pm 2.0 \times 10^3$
