# Supplementary figures and images for: Enhancing Stability and Bioavailability of Peptidylglycine Alpha-Amidating Monooxygenase in Circulation for Clinical Use
Source: Biomolecules. 2025 Feb 4;15(2):224. doi: 10.3390/biom15020224 (PMC11853079; doi:10.3390/biom15020224)

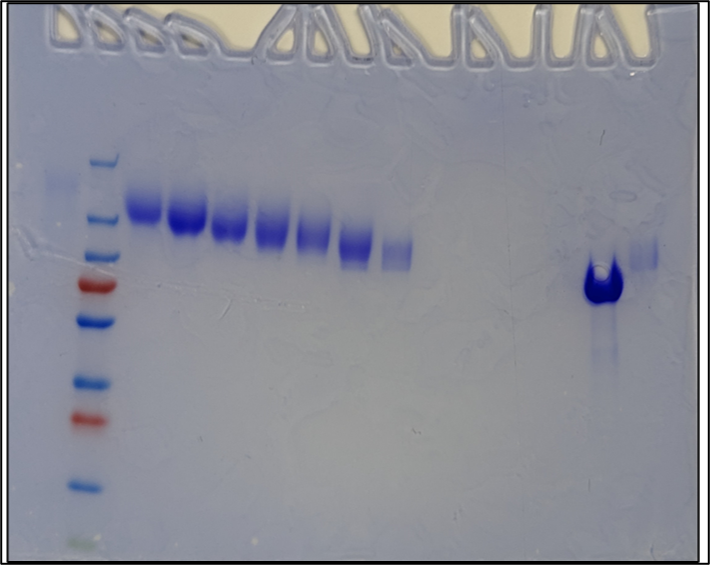

Supplement: Supplementary file 1 [file biomolecules-15-00224-s001.zip › biomolecules-3405411-supplementary/Supplementary File S1/SDS_Gel.png]

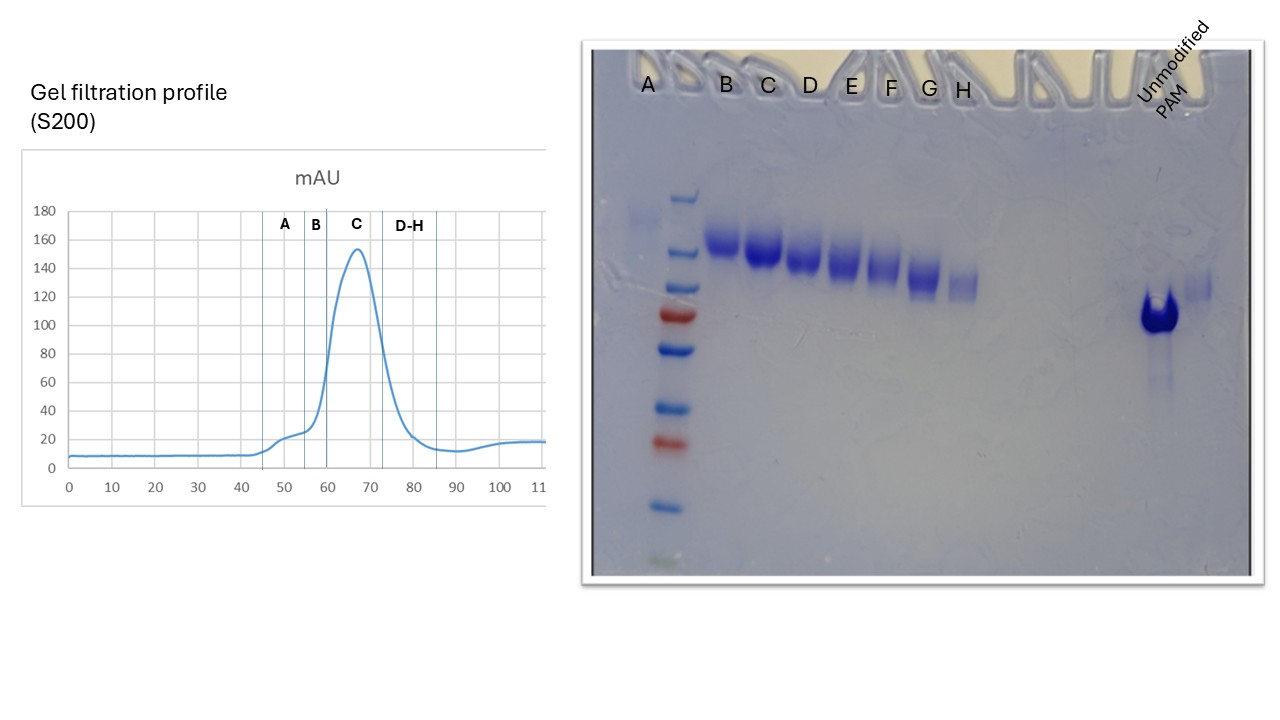

Supplement: Supplementary file 1 [file biomolecules-15-00224-s001.zip › biomolecules-3405411-supplementary/Supplementary File S1/SDS_Gel_labled.jpg]
